# Coupling logistic model tree and random subspace to predict the landslide susceptibility areas with considering the uncertainty of environmental features

**DOI:** 10.1038/s41598-019-51941-z

**Published:** 2019-10-25

**Authors:** Xiangang Luo, Feikai Lin, Yihong Chen, Shuang Zhu, Zhanya Xu, Zhibin Huo, Mengliang Yu, Jing Peng

**Affiliations:** 1 School of Geography and Information Engineering, China University of Geosciences, Wuhan, 430074 China; 2 Class 3 Grade3, Wuhan No.11 High School, Wuhan, 430030 China; 3 Institute of Hydrogeology and Environmental Geology, Chinese Academy of Geological Sciences, Hebei, China; 4 China Institute of Geo-Environment Monitoring, China Geological Survey, Beijing, China

**Keywords:** Environmental impact, Natural hazards

## Abstract

Landslide disasters cause huge casualties and economic losses every year, how to accurately forecast the landslides has always been an important issue in geo-environment research. In this paper, a hybrid machine learning approach RSLMT is firstly proposed by coupling Random Subspace (RS) and Logistic Model Tree (LMT) for producing a landslide susceptibility map (LSM). With this method, the uncertainty introduced by input features is considered, the problem of overfitting is solved by reducing dimensions to increase the prediction rate of landslide occurrence. Moreover, the uncertainty of prediction will be deeply discussed with the rank probability score (RPS) series, which is an important evaluation of uncertainty but rarely used in LSM. Qingchuan county, China was taken as a study area. 12 landslide causal factors were selected and their contribution on landslide occurrence was evaluated by ReliefF method. In addition, Logistic Model Tree (LMT), Naive Bayes (NB) and Logistic Regression (LR) were researched for comparison. The results showed that RSLMT (AUC = 0.815) outperformed LMT (AUC = 0.805), NB (AUC = 0.771), LR (AUC = 0.785). LSM of Qingchuan county was produced using the novel model, it indicated that landslides tend to occur along with the fault belts and the middle-low mountain area that is strongly influenced by the large numbers of human engineering activities.

## Introduction

Landslide is a geological natural disaster usually caused by rainfall, snowmelt, groundwater, earthquake or human activities^1^. It has affected more than 5.5 million people since 1950. In China, the economic losses caused by landslide can reach 28.5 billion dollars every year. Due to the destructive impacts of landslides and their consequences, researchers have long attempted to improve disaster prevention and management, optimize region planning by delineating landslide susceptible areas.

Landslide susceptibility mapping (LSM) is usually regarded as an essential part of the landslide prediction^2^. Generally, methods of LSM can be categorized into physically-based models, statistics-based analysis, and machine learning techniques. Physically-based models are less applied since they require various geographical, geological and hydrometeorological data as well as detailed mathematics and physics equations that simulating the dynamic process of landslide mechanism. As a result, past decades witness the development of statistical models for landslides susceptibility analysis. Statistical models assume that factors and landslide in the past are the same or similar to those in the future^3^. The choice and classification of landslide conditioning factors directly affect the result of LSM. Traditional statistical models, like logistic regression (LR), predefine an appropriate fitting structure and then parameterize it using historical disaster data^4,5^. Machine learning methods are powerful data-driven algorithms, which learn the nonlinear relationship between landslide occurrence and environmental factors. The advantages of machine learning models lie in allowing any scale and type of independent variable, no normal assumption, strong nonlinear fitting capabilities, and many open-source implementations. Various machine learning models for assessing landslide susceptibility like artificial neural network (ANN), classification and regression trees (CART), support vector machine (SVM), neuro-fuzzy (NF), native Bayes (NB), and extreme learning machines (ELM) are carried out intensively in recently years^6–12^.

However, although these machine learning classifiers have been widely used for landslide study, sometimes, the single classifier could perform well in one region but works badly in another. It indicates that the accuracy of the single classifier is variable^2^. Therefore, ensemble methods have been employed in classification to minimize the limitations of a single model. Researchers have found that ensemble techniques could boost both recognition precision and prediction ability by integrating multiple classifiers to improve generalization capabilities^13,14^.

Many hybrid models have been applied in landslide prediction research. Kanungo *et al*.^15^ developed a hybrid (ANN-fuzzy logic) model for landslide susceptibility assessment. Chalkias *et al*.^16^ proposed a hybrid model by coupling expert knowledge with statistical analysis. Peng *et al*.^17^ introduced a hybrid approach by combining rough set and SVM while Oh and Pradhan^18^ proposed a hybrid method of neuro-fuzzy for landslide susceptibility zonation. Pham *et al*.^19^ ensembled the Multiple Perceptron Neural Networks and ensemble frameworks (AdaBoost, Bagging, Dagging, MultiBoost, Rotation Forest, and Random SubSpace) and compared performance of them. Bui *et al*.^13^ represented a novel soft computing approach that combined the fuzzy k-nearest neighbor algorithm (fuzzy k-NN) and the differential evolution (DE) optimization for spatial prediction of rainfall-induced shallow landslides. These hybrid models have all been proved to perform well for landslide susceptibility mapping.

To quantify the certainty of input parameters, Monte Carlo simulations have been used to assess the propagation effect of the uncertainties in digital elevation models and landslide inventory^20–22^. Monte Carlo simulations require a large number of iterations, alternatively, random subspace (RS) is a learning framework which divides high-dimensional environmental features dataset into several low-dimensional subspaces randomly. Multiple classifiers are trained on these subspaces and the results are combined to produce final decision rule^23^. By this way, the uncertainty introduced by input features is considered. Therefore, it is chosen to be a suitable ensemble framework to construct the prediction model with higher confidence degree.

On the other hand, overfitting is an important reason for the decline in the prediction accuracy of the basic model. Noisy hidden in landslide causal factors tend to affect the performance of the model. So, solving the overfitting problem is also a key measure to improve the accuracy of LSM. Logistic Model Tree (LMT)^24^ combines the logistic regression model and decision tree, regarded as one of the most outstanding methods. A logistic tree identifies a set of optimal values of input parameters assigned to each model based on the relative belief in their accuracy. It is opposed to the deterministic model where all weight is put on a single set of parameter values. But the direct application of the LMT approach can still be very time-consuming for regional mapping of landslide hazards. From the above analysis, by combining the method of RS and LMT, the hybrid model will have the advantages of decreasing uncertainty, improving accuracy and lower time-consuming. In previous landslide susceptibility studies, some researchers have completed similar works. Shirzadi *et al*.^9^ combined RS and NBT to construct a novel model and Pham *et al*.^25^ proposed a hybrid model based on RS and CART. Their models have improved a lot than single classifier NBT and CART and they are both promising methods for landslide susceptibility mapping. But Bui *et al*.^26^ compared five landslide models and proved that LMT was a better model for producing LSM. So, LMT may be a better choice than NBT or CART. On the other hand, Chen *et al*.^27^ ensembled bivariate statistical approach and LMT and Truong *et al*.^28^ constructed the model based on Bagging and LMT, their models were also proved appropriate. Compared with them, RS has the advantage of decreasing uncertainty, it is also worth trying.

The main purpose of this research is to propose a hybrid intelligent approach RSLMT based on RS and LMT to produce more accurate LSM. What’s important, besides the common receiver operating characteristic (ROC) curve, the uncertainty of prediction will be deeply discussed with the rank probability score (RPS) series, which is an important evaluation of uncertainty but rarely used in LSM. The hybrid method is firstly proposed in Landslide susceptibility research, and it will be compared to common methods like LMT, LR, NB. This study attempts to decrease the uncertainty and produce more reliable data concerning LSM, which can support the land development and decision-making process.

## Study Area and Data Resource

### Study area

**Figure 1:**
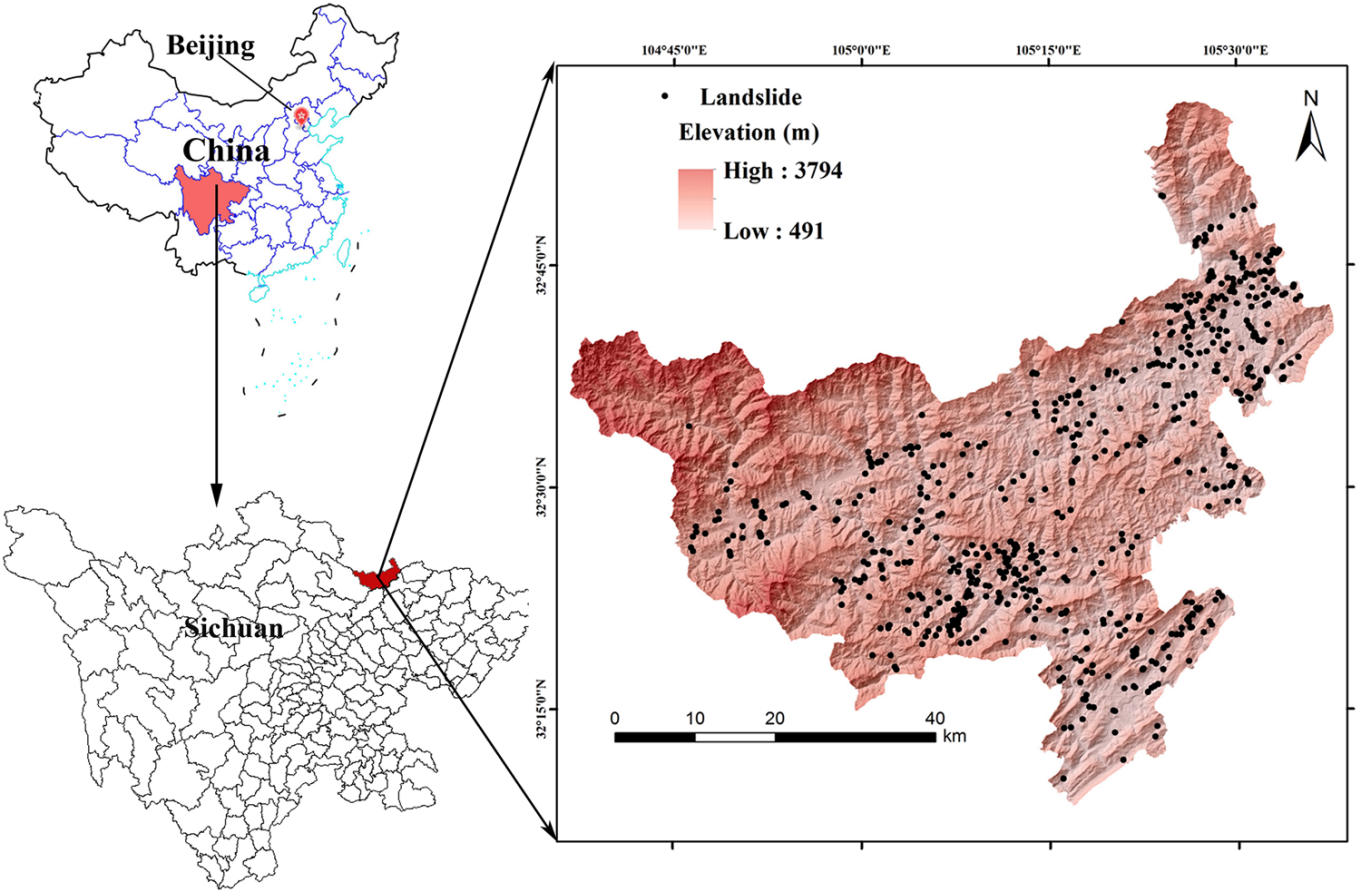
The study area, Qingchuan County in Sichuan Province China, generated by Arcgis version 10.2 in Windows (https://developers.arcgis.com).

Qingchuan County is located in the northern part of Sichuan Province between 32°12′ and 32°56′ north latitude, 104°36′ and 105°38′ east longitude, with the area of 3271 km^2^. Figure 1 shows the study area. This area has very complicated geological and tectonics conditions. From the Cambrian to the Jurassic period, there were various sediments (limestone, sandstone, and conglomerate), magma (granite) and metamorphic rocks (shale, schist, gneiss). Sedimentary deposits and quaternary loess are widely exposed to dense fault structures. Seismic activities have occurred frequently, including the Wenchuan earthquake (2008) and Lushan earthquake (2013). The two earthquakes caused enormous loss, bring great threats to post-disaster reconstruction.

Qingchuan County has two active faults, Pingwu - Qingchuan fracture and Yingxiu - Beichuan fault, Longmenshan fault zone, 60–70 - NW is oblique thrust fault. The terrain of Qingchuan County is characterized by low northwest and high southeast. The central part has an altitude of 1200–1800 m. The average slope is 38°, the maximum slope is 80°, and the 73.9% of the area has a slope of more than 25°. Qingchuan County has a subtropical monsoon climate with mild summer temperatures and southwesterly winds. The study area has abundant rainfall and the annual average rainfall is 1022 mm. 55% of the rainfall occurs in June to September every year^29^.

### Landslide inventory

The landslide inventory is an essential part of LSM, it includes historical landslide data and other related information like geological data, meteorological conditions, and topographical data^30^. The landslide inventory of Qingchuan was extracted from the geological disaster database which was provided by China Geological Survey (http://www.cgs.gov.cn/). The original scale of landslides is more than 1400. Considering the dramatic changes in the geographical environment, 640 landslides of the same type occurred in the latest 2009–2013 were selected in the study. In landslide susceptibility assessment, negative samples (non- landslide) are as important as positive samples. So, 629 non-landslide points were selected in the study area randomly to construct a data set. All of them were processed by ArcGIS 10.2. Due to most of the landslides have a small influence region, all landslides were simplified as points, represented by a pixel (30 m * 30 m). In this study, 70% of the data was chosen randomly for model training, and the other data was used for the verification.

### Landslide causal factors

**Figure 2:**
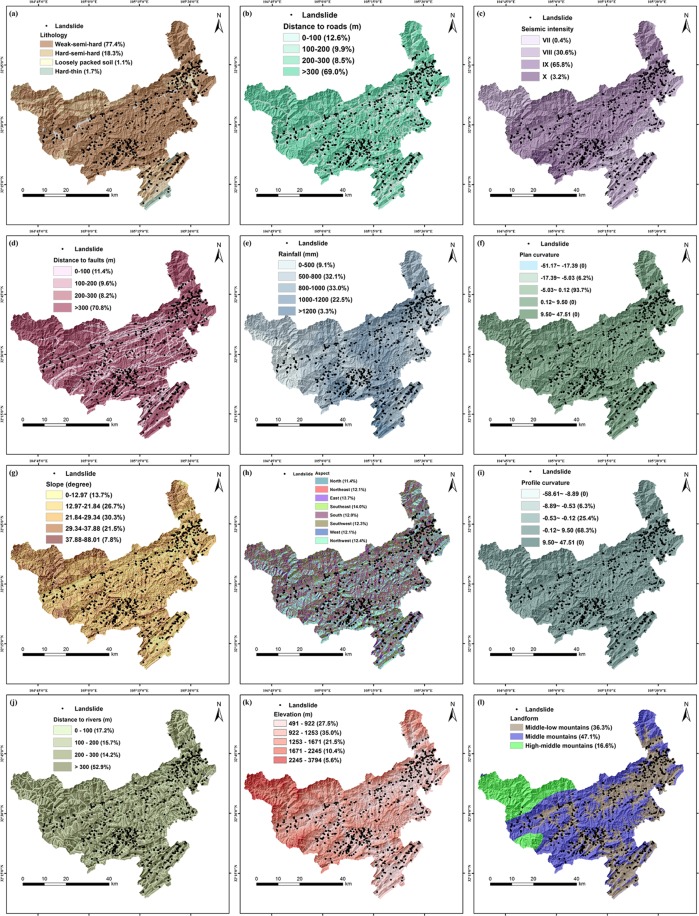
Maps of landslide causal factors. (**a**) Lithology. (**b**) Distance to roads. (**c**) Seismic intensity. (**d**) Distance to faults. (**e)** Rainfall. (**f**) Plan curvature. (**g**) Slope. (**h**) Aspect. (**i**) Profile curvature. (**j**) Distance to rivers. (**k**) Elevation. (**l**) Landform, generated by Arcgis version 10.2 in Windows (https://developers.arcgis.com).

How to select appropriate landslide causal factors is an essential issue in landslide susceptibility mapping. There is no standard answer to this question until now^31^, since the cause of the landslide varies over the different region. Based on the analysis of local geological environment characteristics and relevant researches, we selected twelve landslide causal factors in this study. These factors could be divided into two categories: (1) internal factors, which are related to geology and topography, such as elevation, profile curvature, slope, plan curvature, distance to faults, aspect, distance to rivers, landform and lithology; (2) external factors, which usually cause landslides such as rainfall, distance to roads and seismic intensity. Moreover, these factors are reclassified into various categories (Table 1) for the convenience of landslide susceptibility analysis and avoiding the imbalance of categorical magnitudes. Each value represents a group of data with similar characteristics. The numeric values are discrete, representing different classes. Nature breaks was used for classification which is a method that maximizes the differences between classes and minimizes data skew in each class. The elevation, slope, aspect, and curvature were extracted from DEM with 30 m spatial resolution, which could be downloaded from Geospatial data cloud (www.gscloud.cn). The roads, rivers, and landform were extracted from the Qingchuan County topographic map. The rest of the geological meteorological factors were provided by China Geological Survey (http://www.cgs.gov.cn/). All factors were processed using ArcGIS 10.2 and eventually converted to a raster format of the same resolution as DEM for further analysis. They are shown in Fig. 2.

**Table 1:** Landslide causal factors with their classes and quantitative value.

Factors	Classes	Value	Factors	Classes	Value
Lithology	Weak-semi-hard	1	Profile curvature	−58.61–8.89	1
	thin-medium			−8.89–0.53	2
	phyllite			−0.53–0.12	3
	schist			−0.12–9.50	4
	slate			9.50–47.51	5
	metamorphic		Plan curvature	−51.17–17.39	1
	sandstone			−17.39–5.03	2
	Hard-semi-hard	2		−5.03–0.50	3
	medium-thick layered limestone			−0.50–8.98	4
	Dolomitic limestone			8.98–53.88	5
	dolomite		Slope (degree)	0–12.97	1
	debris			12.97–21.84	2
				21.84–29.34	3
	Loosely packed soil	4		29.34–37.88	4
	Hard-thin layered quartz sandstone	5		37.88–87.01	5
	siltstone		Aspect	North	1
	conglomerate			Northeast	2
	mudstone			East	3
Rainfall (mm)	0–500	1		Southeast	4
	500–800	2		South	5
	800–1000	3		Southwest	6
	1000–1200	4		West	7
	>1200	5		Northwest	8
Seismic intensity	VII	7	Distance to faults (m)	0–100	1
	VIII	8		100–200	2
	IX	9		200–300	3
	X	10		>300	4
Landform	Middle-low mountains	1	Distance to rivers (m)	0–100	1
	Middle mountains	2		100–200	2
	High-middle mountains	3		200–300	3
Elevation (m)	491–922	1		>300	4
	922–1253	2	Distance to roads (m)	0–100	1
	1253–1671	3		100–200	2
	1671–2245	4		200–300	3
	2245–3794	5		>300	4

## Methodology

### Outline of RSLMT for LSM

RSLMT for LSM is carried out as follows.

**Figure 3:**
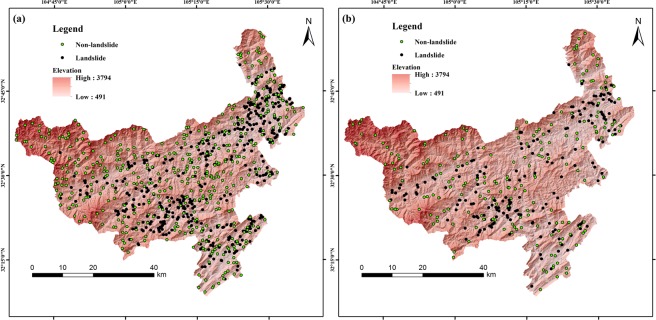
Training data and validation data. (**a**) Training data. (**b**) Validation data, generated by Arcgis version 10.2 in Windows (https://developers.arcgis.com).
2.Model building. Firstly, use the ReliefF method to rank the contributions of landslide causal factors, the factors with lower contributions were sequentially removed. The proposed RSLMT model and comparison models were built in Weka 3.9 software, to achieve the best performance, parameter optimization was performed.
Model Verification. Use area under receiver operating characteristic (AUC) and above statistical index to compare the performance between the new model and other models. Perform uncertainty analysis and chi-square test.
Landslide susceptibility mapping. The best performed model was selected for making landslide susceptibility map of Qingchuan county. The map was graded according to the landslide susceptibility index. Analysis the distribution characters of landslides and explore the cause of landslides.

Data extraction and preprocessing. 640 landslide points, 629 non-landslide points, and 12 landslide causal factor layers were extracted, and then these factors were quantified and classified. 70% of the data was chosen for the training model and the rest was used for validation. Training and validation data were shown in Fig. 3.

**Figure 4:**
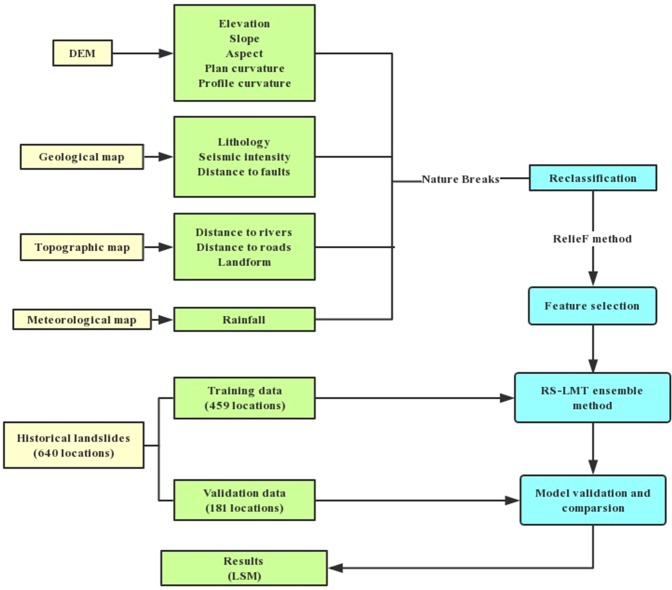
Outline of RSLMT after Pham *et al*.^25^, 2018 with modifications, which has been authorized by Elsevier.

The outline of the study is shown in Fig. 4.

### ReliefF

ReliefF is a feature selection algorithm which was developed by Kononenko^32^, it is an extension of Relief. ReliefF first resamples the instance multiple times and then estimates the values of the attribute by considering the values of specific attributes from the most recent instance of the same and different classes. It will remove the factors that have lower average merit (AM) from the original dataset because these factors are considered less or even no help to the prediction of landslides.

### Logistic model tree

LMT is a combination of logistic regression model and C4.5 decision tree^33^, it uses information gain to spilt and LogitBoost algorithm to produce logistic regression model at every tree node. Classification and regression tree^34^ is used for pruning to prevent over-fitting.

The LogitBoost algorithm uses additive logistic regression with each class *C*_*i*_ having a least squares fit as follows^35^:

1 $${L}_{C}({\rm{x}})=\mathop{\sum }\limits_{i=1}^{n}{\beta }_{i}{x}_{i}+{\beta }_{0}.$$

Linear logistic regression method can be used to calculate posterior probability in leaf nodes^25^,

2 $$P(C|x)=\frac{\exp ({L}_{C}(x))}{\mathop{\sum }\limits_{C^{\prime} }^{D}\exp ({L}_{C^{\prime} }(x))},$$

*D* is the number of classes.

### Random subspace

Random Subspace (RS) is a popular integrated learning approach proposed by Ho^36^. It divides the original feature set into several subsets containing partial features. randomly and trains multiple classifiers on these feature subspaces^37^, so it can improve the classification accuracy [10, 16, 30]. It is especially good at dealing with overfitting problems^24^. The detailed description of RS is as follows.

Suppose the training data are $$X=({X}_{1},{X}_{2},\mathrm{...},{X}_{{\rm{i}}}),(i=1,2,\mathrm{...},n)$$ and $${X}_{i}$$ is a *p*-dimensional vector, *p* is the number of features. Then an *r*-dimensional vector $${\hat{X}}_{i}$$ is carried out from a *p*-dimensional vector $${X}_{i}$$$$(r < p)$$. *r*-dimensional random subspace can be described as

3 $$\hat{X}=[\begin{array}{cccc}{\hat{X}}_{11} & {\hat{X}}_{21} & \mathrm{...} & {\hat{X}}_{{\rm{n1}}}\\ {\hat{X}}_{12} & {\hat{X}}_{22} & \mathrm{...} & {\hat{X}}_{n2}\\ \mathrm{...} & \mathrm{...} & \mathrm{...} & \mathrm{...}\\ {\hat{X}}_{1r} & {\hat{X}}_{21} & \mathrm{...} & {\hat{X}}_{nr}\end{array}].$$

In the next step, repeat this selection several times, and some lots of *r*-dimensional random subspaces could be obtained. Finally, construct classifier *C*(*x*) in every subspace $\hat{X}$, and combine the results of these classifiers with final decision rule by a simple majority vote. The final decision rule is as follows,

4 $$\beta (x)={\rm{\arg }}\,{\rm{\max }}\,\mathop{\sum }\limits_{b}^{\delta }{\delta }_{{\rm{sgn}}}({C}^{b}(x)),y;y\in \{-1,1\},$$

where ${\delta }_{i,j}(i=1,2,\mathrm{...},n,j=1,2,\mathrm{...},r)$ is the Kronecker symbol, and $y\in \{1,-1\}$ is a class label (landslide or non-landslide).

### Statistical index

Statistic index involves accuracy, precision, *F*-measure, accuracy, specificity, and recall^25,38^. Accuracy is the percentage of samples that are correctly classified in the total sample, the higher the accuracy, the better the classifier. Precision is the proportion of samples that are predicted to be positive in the positive sample set. Recall is the proportion of correct classification in all positive samples. Specificity is opposite to the recall, it is the proportion of true classification in all negative samples. And usually precision and recall are conflicting, the *F*-measure is a weighted average of precision and recall which is considered more balanced indicator.

5 $$Accuracy=\frac{TP+TN}{TP+FP+TN+FN},$$

6 $$precision=\frac{TP}{TP+FP},$$

7 $${Recall}=\frac{TP}{TP+FN},$$

8 $$Specificity=\frac{TN}{TN+FP},$$

9 $$F-measure=\frac{2\times TP}{2\times TP+FP+FN},$$

where *TP* (true positive) and *TN* (true negative) are the number of samples that are correctly classified. *FP* (false positive) and *FN* (false negative) are the number of samples that are incorrectly classified.

Traditional statistical indexes mentioned above are usually used to assess the classification results right or not, without considering the uncertainty of the classification. The rank probability score (RPS) which was proposed by Epstein^39^ is a suitable measure for the uncertainty of the classification. It calculates the cumulative error between the predicted category and the actual category. For *K* categories, the RPS is defined as follows,

10 $$RPS=\mathop{\sum }\limits_{k=1}^{K}{({F}_{k}-{O}_{k})}^{2}={({\bf{F}}-{\bf{O}})}^{2},$$

where *F* and *O* are cumulative predicted and actual vectors. ${F}_{k}$ and ${O}_{k}$ are defined as $\mathop{\sum }\limits_{i=1}^{k}{F}_{i}$ and $\mathop{\sum }\limits_{i=1}^{k}{O}_{i}$, ${F}_{i}$ is the forecasted probability that the point is classified into *i* category. ${O}_{i}$ is the actual classification, if the category is *i*, ${O}_{i}=1$, if not, ${O}_{i}=0$. The closer the RPS is to 0, the better the classification result. The RPS of the reference model is calculated as same as that of the predictive model. For the reference model, we chose the widely used historical sample point analysis, in this way, the probability that the point is classified into *i* category is the ratio of the number of historical points actually belong to *i* category to the number of total points.

In addition to RPS, RPS score (RPSS)^40^ is also used to estimate the uncertainty of models. It could measure how well the prediction model improves relative to the reference model. For RPSS, the maximum value 1 represents a perfect model, whereas a value of 0 represents the model is no better than the reference model, a negative value indicates the model performs worse than the reference model. RPSS is calculated as follows:

11 $$RPSS=1-\frac{\overline{RP{S}_{m}}}{\overline{RP{S}_{r}}},$$

where $\overline{RP{S}_{m}}$ and $\overline{RP{S}_{r}}$ are average RPS values of the predictive model and reference model. A positive RPSS value indicates that the predictive model is better than the reference model.

## Results

### Landslide causal factors analysis

ReliefF was implemented on the prepared twelve factors. The result indicated that seismic intensity, distance to faults, distance to rivers, profile curvature had lower contribution. To find the factors that should be removed, these factors were removed one by one in the RSLMT model using 10-fold cross-validation. AUC values are 0.771, 0.774 and 0.768 when removing the lowest one, two, three factors, respectively. The performance is better when removing two factors, profile curvature and distance to rivers. The variance inflation factor (VIF) and tolerance^38,41^ were used to examine the multicollinearity within the remaining ten factors. A VIF above 5 or tolerance of less than 0.2 indicates the existence of multiple collinearity^42^. As shown in Table 2, the minimum tolerance among the factors is 0.257, and the highest VIF is 3.891. There is no multicollinearity between these factors. So, ten factors were left for further analysis.

**Table 2:** Multicollinearity of the causal factors.

Landslide causal factors	Multicollinearity statistics	
	Tolerance	VIF
Rainfall	0.896	1.116
Seismic intensity	0.885	1.130
Lithology	0.846	1.182
Landform	0.259	3.860
Distance to faults	0.962	1.039
Distance to roads	0.736	1.359
Elevation	0.257	3.891
Plan curvature	0.981	1.019
Slope	0.853	1.172
Aspect	0.970	1.031

### Model validation and comparison

**Figure 5:**
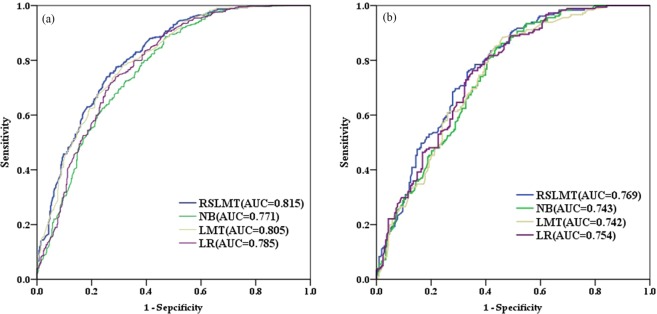
AUC of the models. (**a**) Training data. (**b**) Validation data.

Using the training data, RSLMT, NB, LR, LMT were constructed and the performance of these models was evaluated by validation data. After the trial-and-error process, the optimum parameters used by these models are shown in Table 3. AUC results with training data and validation data are shown in Fig. 5, and results of statistical index are shown in Table 4.

**Table 3:** The calculated parameters of algorithms utilized in this study.

Algorithm	Parameters					
RSLMT	Minimum subspace	0.5	Seed	1	Iteration	8
	Execution slots	1	Instances in node	21	LogitBoost iterations	7
NB	/					
LR	Maximum number of iterations	8	Ridge value in the log-likelihood	10^−8^		
LMT	Minimum of instances in node	15	LogitBoost iterations	3	Weight trimming value	0.0

**Table 4:** Performance of models using training and validation data.

Statistic index	RSLMT		NB		LR		LMT	
	T	V	T	V	T	V	T	V
Accuracy	0.738	0.697	0.703	0.686	0.716	0.694	0.727	0.674
Precision	0.715	0.639	0.682	0.630	0.714	0.653	0.713	0.625
Recall	0.826	0.801	0.808	0.790	0.760	0.729	0.800	0.746
Specificity	0.641	0.606	0.589	0.596	0.667	0.663	0.648	0.611
F-measure	0.766	0.711	0.740	0.701	0.736	0.689	0.754	0.680

T = training data; V = validation data.

AUC on training data represents the goodness of fit of model^14^. Among these models, RSLMT model (AUC = 0.815) shows the best goodness of fit, followed by the LMT model (AUC = 0.805), the LR model (AUC = 0.785), the NB model (AUC = 0.771). And the AUC on validation data represents prediction abilities of models, the results show that the prediction ability of RSLMT model (AUC = 0.769) is the best and that of LMT model (AUC = 0.742) is the worst, the AUC of NB model and LR model are 0.743 and 0.754. For statistical index in training data, the accuracy of RSLMT model is the highest (0.738), followed by the LMT (0.727), LR (0.716), NB (0.703) models. For validation data, the accuracy of RSLMT model is still the highest (0.697), followed by the LR (0.694), NB (0.686), LMT (0.674) models. LR model has the highest precision (0.653), followed by the RSLMT (0.639), NB (0.630), LMT (0.625) models. For more, the RSLMT model has also the highest F-measure and recall among the models. In summary, it can be inferred that RSLMT has the best performance both in training and validation data.

The uncertainty analysis results of these models are shown in Table 5. It can be observed that the RPS of RSLMT is the smallest, showing a smaller range of uncertainty, followed by LR (RPS = 0.203), LMT (RPS = 0.207), NB (RPS = 0.231) models. And the RSLMT model also has the highest RPSS value, indicating that the RSLMT model has the biggest improvement compared to the reference model. Moreover, statistical differences between these models were tested using Chi-Square. In case the Chi-Square value is greater than 3.841 and the significance level value (*p*) is smaller than 0.05, the assumptions of two significantly different models are correct, so the difference of these models is statistically significant^43^. The Chi-Square test results of the RSLMT model compared with others are shown in Table 6. It could be found that all Chi-Square values exceed 3.841 and all *p*-values are less than 0.5. It means that the performance of RSLMT is significantly different from other models and the RSLMT model is comparable to other models.

**Table 5:** The RPS and RPSS values of the models.

Model	$\overline{{\boldsymbol{RP}}{{\boldsymbol{S}}}_{{\boldsymbol{m}}}}$	$\overline{{\boldsymbol{RP}}{{\boldsymbol{S}}}_{{\boldsymbol{r}}}}$	*RPSS*
RSLMT	0.196	0.286	0.315
NB	0.231	0.286	0.192
LMT	0.207	0.286	0.276
LR	0.203	0.286	0.290

**Table 6:** Performance of the RSLMT model compared to other models using Chi-Square test.

Comparative pairs	Chi-square values	*p*-value
RSLMT vs. NB	604.063	0
RSLMT vs. LR	539.001	0
RSLMT vs. LMT	543.939	0

### Landslide susceptibility mapping

**Figure 6:**
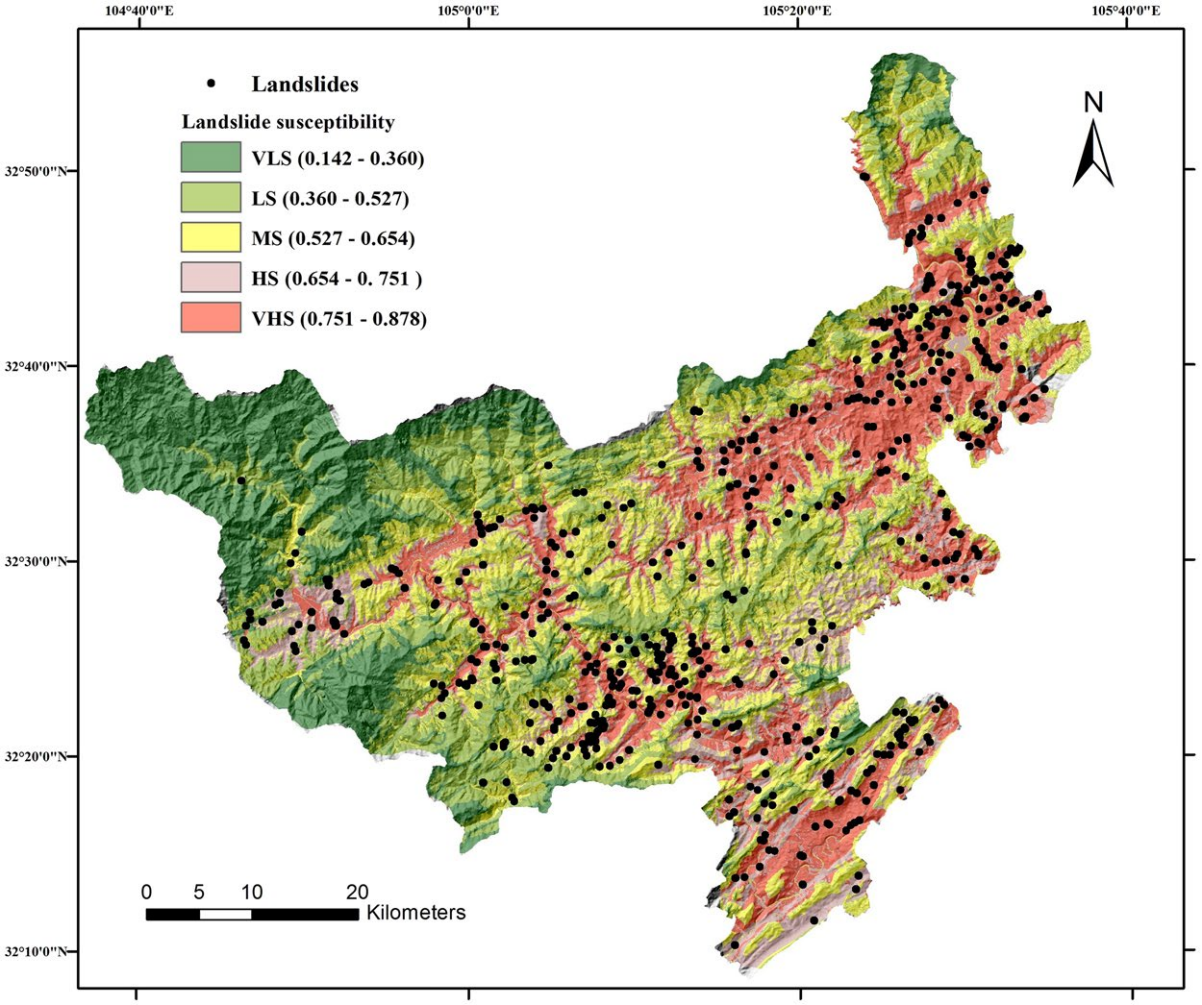
Landslide susceptibility map in Qingchuan county using the RSLMT model, generated by Arcgis version 10.2 in Windows (https://developers.arcgis.com).

After the validation and comparison, the best performed model RSLMT were used to produce LSM. Study area was conversed to a raster map using ArcGIS 10.2 with 30 m resolution. Then, landslide susceptibility index (LSI) was calculated as the probability of landslide occurrence using RSLMT model. Each pixel was assigned unique LSI. Finally, LSM was classified into 5 categories through Geometrical Interval (GI) method^44^, very low susceptibility (VLS), low susceptibility (LS), moderate susceptibility (MS), high susceptibility (HS), very high susceptibility (VHS). LSM is shown in Fig. 6.

To estimate the precision of the LSM, relative landslide density was calculated. The relative landslide density is the ratio of the percentage of landslides in the sensitive area to the total number of landslides and the percentage of the sensitive area to the total study area. The relative landslide density is shown in Table 7.

**Table 7:** Relative landslide density of each class in LSM.

Classes	Percentage of area (%)	Percentage of landslide points (%)	Relative landslide density
VLS	18.35	0.47	0.0256
LS	20.11	5.78	0.2874
MS	24.98	20.16	0.8070
HS	15.32	21.56	1.4073
VHS	21.24	52.03	2.4496

From the table, it can be found that the VHS area which occupies only 21.24% area has the 52.03% of landslides, however, only 0.47% of landslides are distributed in the VLS area which occupies the 18.35% of the area.

## Discussion

The selection of landslide causal factors is an essential issue in landslide modeling^10^. Based on the comprehensive analysis, twelve factors were selected. Then these factors were reclassified using Nature Breaks which is applied in many studies and proved to have no effect on the results. The ReliefF model was used for computing the contribution of factors to the occurrence of the landslide. After sequentially removing the factors with lower average merits using RSLMT model, it had been found that RSLMT had the best performance removing distance to rivers and profile curvature. So, it is necessary to select factors before applying ensemble learning method, even if RS has the ability to reduce dimensions. Pham *et al*.^25^ used the LSVM to optimize input data and found the same conclusion. Some factors could have a negative impact on some feature subspace, and reduce the accuracy of the model.

In the past, scholars mainly used statistical techniques or machine learning methods to make landslide prediction. These single classifiers performed well in many regions. In recent years, hybrid models are beginning to be applied in this field. Many review papers indicate that hybrid models are more efficient than traditional individual classifiers like SVM, LR, DT for landslide spatial prediction because hybrid model could integrate multiple classifiers to improve generalization capabilities^13^. Moreover, Pham *et al*.^19^ stated that hybrid techniques – in certain conditions – can improve the performance of individual classifiers for landslide susceptibility analysis.

In this paper, a novel hybrid model called RSLMT was proposed, and it was used to produce LSM based on the following assumptions: the landslide mechanism is the same for all the landslides in the test set; there is no spatial heterogeneity in the relationship between conditional factors and landslide susceptibility^45,46^; there are no mutual relationships between conditioning factors; the mechanism responsible for past landslides in the study area will introduce future landslides; the output LSM presents only the predicted spatial distribution of landslides and not its temporal probability.

Comparison between the proposed model and other excellent machine learning classifiers had been done. AUC was used to estimate the performance of models in both training and validation data. The RSLMT model outperformed the LMT, LR, NB models on training data, it indicated the better goodness of fit. For the validation data, the RSLMT model had the best AUC of 0.769, followed by the LR, NB, LMT models. The results can prove that the RS method can improve the performance of individual classifier significantly. This could be found in many studies^9,19,25^. Abedini *et al*.^47^ ensembled Bayesian Logistic Regression (BLR) and ensemble method RS, Adaboost, Multiboost and Bagging and compared the accuracy of these models, they found that RS-BLR performed best. It proved that the RS model is one of the best ensemble methods. On the other hand, it is worth noting that the LMT model had the second-highest AUC on training data but had the worst prediction ability on validation data just as the finding of Bui *et al*.^26^. Serious overfitting problem existed in LMT. And the RSLMT performed well both on training and validation data, it could be inferred that RS method can avoid the overfitting problem of the classifier. It can be found that RSLMT has the advantages of decreasing uncertainty, improving accuracy and reducing time-consuming compared with other models and it can be a promising method for landslide susceptibility mapping, and it can also be applied to other landslide-prone regions.

Although the RSLMT outperformed than other models in this study, there is still room for improvement. The model performance was only demonstrated in one region, which cannot prove its adaptability. Therefore, the model performance in regions featured with different geological environment characteristics needs to be further studied and verified when data are available.

From the produced landslide susceptibility map in Qingchuan county, it can be found that landslides occurred mostly in the VHS area. Comparing the LSM with faults map, it is obvious that landslide tends to be distributed along with the fault belts, especially along the Yingxiu-Beichuan fault. And comparing the LSM with landform and roads map, middle-high mountains area is usually safer because of the stable geologic environment. On the contrary, the middle-low mountains have the highest susceptibility index due to a large number of human engineering activities. Reducing human damage to the geological environment may be an important measure for preventing the occurrence of the landslide.

## Conclusion

The prediction of landslide occurrence is important, it is vital to propose new models to enhance the ability to predict. In this paper, a novelty ensemble learning models based on the random subspace and logistic model tree namely RSLMT model was developed for producing landslide susceptibility map of Qingchuan county, China. In the research, 12 landslide causal factors were chosen based on relevant analysis and local geological environment characteristics. After the selection with ReliefF method, distance to rivers and profile curvature were removed because of lower contribution to landslide occurrence. Then, the AUC and a set of statistical indexes were used to evaluate and compare RSLMT model with NB, LR, LMT. Results show that RSLMT model has the best performance. Finally, LSM was produced by RSLMT model and classified into 5 categories. This map will contribute to land use, hazard management, and decision making.

## Data Availability

The data used to support the findings of this study are available from the corresponding author upon request.
